# Treatment-seeking behaviours of patients with tungiasis in endemic areas of Homa Bay County, Kenya: a mixed-methods study

**DOI:** 10.1186/s41182-024-00639-8

**Published:** 2024-10-22

**Authors:** Kana Suzuki, Asiko Ongaya, Gordon Okomo, Muuo Nzou, Evans Amukoye, Yasuhiko Kamiya

**Affiliations:** 1 School of Tropical Medicine and Global Health, Nagasaki University, Nagasaki, Japan; 2 Kenya Medical Research Institute, Nairobi, Kenya; 3 Ministry of Health Homa Bay County, Homa Bay, Kenya

**Keywords:** Tungiasis, Treatment-seeking behaviour, Kenyan national policy guidelines

## Abstract

**Background:**

Tungiasis, an ectoparasitic disease caused by sand fleas, causes suffering to millions of people in the tropics. Although the Kenyan National Policy Guidelines list tungiasis treatments as including disinfectants, flea repellents, and botanical oil, the insufficient knowledge and financial constraints of affected communities have led to neglect and inappropriate self-treatment. Current reports show insignificant progress on educational activities at the community level. Therefore, we investigated community residents’ treatment-seeking behaviour concerning tungiasis, using an endemic area of Kenya as the research setting.

**Methods:**

A cross-sectional mixed-methods design was employed. Quantitative data were collected from the participants—410 adults who had experienced tungiasis—using a questionnaire, while qualitative data were collected from 20 older adults to 10 medical staffs using semi-structured individual interviews. The study was conducted in two sub-counties of Homa Bay County, Kenya.

**Results:**

Factors significantly correlated with using non-guideline-listed treatments for tungiasis were ‘not knowing the causse of tungiasis’, ‘not seeking treatment from healthcare facilities and traditional healers’, and ‘wait and see to prevent infection in non-affected members’. The interviews with the older adults revealed 19 self-treatment options for tungiasis, and 40% of the participants opted for self-removal using sharp objects. Only two of these treatments were listed in the guidelines. The most frequently mentioned reason for using a self-treatment option was ‘Someone else’s idea’. The most frequently mentioned reason for choosing the best self-treatment option was ‘Effectiveness’. Interviews with medical staff revealed 11 treatment options; only five of these treatments are listed in the guidelines. The most frequently mentioned reason for selecting/using the treatment was ‘Supply situation’.

**Conclusions:**

Residents’ socioeconomic factors, cultural factors, and access to appropriate treatment, as well as knowledge of medical staff were significant factors that influenced the residents’ tungiasis treatment-seeking behaviours. This study provides feasibility and baseline data to establish an effective, safe, and sustainable treatment for tungiasis.

## Background

Tungiasis is a cutaneous parasitosis caused by the female sand flea, *Tunga penetrans* [1]. *T. penetrans* is distributed in tropical and subtropical regions globally, particularly in the Caribbean, South America, and sub-Saharan Africa, where it is described as a chigoe, jigger, or sand flea infection [2]. Owing to its prevalence in Africa, tungiasis is considered one of the ‘neglected tropical diseases’ (NTDs) by the World Health Organization [3], calling for priority measures to ensure its eradication [4]. Precarious living conditions enhance the multiplication and survivability of sand fleas. Thus, the highest prevalence and disease burden are mainly experienced by the poorest in South American and sub-Saharan African communities [5]. Based on global economic disparities,  ~ 700 million people are at risk of experiencing tungiasis. The burden of tungiasis ranges from 23 to 63% in endemic areas in African countries [3, 4, 6]. In Northeastern Uganda, the prevalence of tungiasis among residents was 63% [6]. Tungiasis presents varied geographical distributions due to a complex number of factors, including human-related, topographical, and environmental aspects that affect disease development.

Nonfertilised *Tunga penetrans* females penetrate into the skin to get blood and remain there until they die in situ after 4–6 weeks [7]. In homes with unsealed earthen floors, the sand flea life cycle can be completed indoors within sleeping areas [8]. When a person sleeps, eggs expelled by the fleas fall directly to the floor. These eggs can remain in place, where the larvae feed on the abundant organic material present. During their off-host stage, *Tunga* *penetrans* typically stay around the host's feet [8]. Eventually, the adult fleas that emerge from their pupae attach to and penetrate the skin when a person places their bare feet on the ground [9]. In tropical environments, three potential hosts for the sand flea's transmission cycle coexist humans, domestic animals, and wildlife. These cycles can partially or completely overlap depending on the local interactions between humans and animals [9].

The main risk factors for tungiasis include poor housing [10, 11], inadequate hygiene [11], low socioeconomic status [12], lack of knowledge of health behaviours [10, 12], and the presence of animals [10, 11, 13]. In Kenya, this disease predominantly affects marginalised people living in resource-poor communities such as rural semi-arid areas and coastal regions. Public health awareness of tungiasis has been growing recently in Kenya due to its impactful role among school-going children and the results from pilot studies conducted in endemic areas. Children with tungiasis have disproportionately high absenteeism and perform worse in school than unaffected pupils [14]. This can be attributed to the constant itching and pain, which makes concentrating on studies difficult [15]. In Homa Bay, Kenya, it is unclear what percentage of absenteeism is due to tungiasis, but Ministry of Health officers have identified severe tungiasis cases (e.g. more than 30 fleas on a single child) at home (JICA grassroots project survey, 2022). If manually and unhygienically extracted, these infection treatments may result in secondary bacterial infection [5] and walking difficulties due to deformity of the toes [3]. Another age group at risk of tungiasis is that of older adults [16]; however, besides one case report [17], no studies have focused on older adults.

Major prevention mechanisms for tungiasis include cleaning the limbs, applying a repellent to the skin, and improving one’s living conditions, such as cementing house floors and wearing shoes [16]. However, such preventions are often not affordable and sustainable for impoverished households in resource-scarce areas. Thus, developing risk-specific prevention and control mechanisms based on comprehensive knowledge of the patterns of tungiasis is essential. Pilot studies have linked high tungiasis prevalence to Nyanza and the central, coastal, western, and Rift Valley regions in Kenya [4, 8, 12, 13]. These specific endemic areas are associated with limited epidemiological understanding, unhygienic conditions, soil factors, animal rearing, and community-specific vulnerabilities [4, 13]. Therefore, community-based preventive measures are essential for the effective and practical implementation of the identified mechanisms. Manual and unhygienic extraction of embedded sand fleas is highly common but has several shortcomings. Surgical extraction of embedded sand fleas is common, but has several shortcomings. People living in endemic areas use safety pins, needles, scissors, a knife, or sharply pointed pieces of wood [5]. These practices are time-consuming, painful, and often result in secondary infections (e.g. tetanus, HIV, and hepatitis B and C). In fact, these practices are time-consuming and painful and are suspected to result in secondary infections; such as tetanus, HIV, and hepatitis B and C. This has been linked to the high prevalence of hepatitis B and C in sub-Saharan Africa [18, 19]. Individuals may also abstain from treatment due to the pain. In case of secondary infection after removal, additional expenses are required for the purchase of antibiotics and anti-tetanus toxoids, increasing the economic burden on poor patients [20].

Kenyan National Policy Guidelines [21] list treatments including disinfectants such as Savlon, potassium permanganate, and hydrogen peroxide, as well as flea repellents such as Deet, Zanzarin, neem and coconut oil preparations, jojoba oil, and aloe vera extract. A mixture of low-viscosity silicone oils (dimeticones) is also strongly recommended as it is a more effective and non-toxic therapeutic drug currently being used in the field [22].

Promoting and increasing intervention studies on tungiasis treatment will enhance the understanding of the disease in relation to the treatment-seeking behaviour of residents in endemic areas. The limitedness of information on prompt therapeutic interventions limits individuals, physicians, and medical staff from selecting effective treatment options. Tendencies to neglect treatment and perform inappropriate self-treatments can be attributed to insufficient knowledge and financial constraints. Although the Kenyan Government Guidelines provide numerous options for treating tungiasis, there is limited evidence on the degree and kind of treatment options received at healthcare facilities. Therefore, this study aimed at providing evidence on knowledge, attitude, and practice (KAP) data, as well as socio-demographic data and treatment-seeking behaviours in the tungiasis-endemic regions of Ndhiwa and Suba South sub-counties, Homa Bay County, Kenya. This study is the first to clarify residents’ treatment-seeking behaviours against tungiasis and the first to provide evidence of tungiasis in Homa Bay County, Kenya.

## Operational definitions

### Guideline-listed treatments

Treatments recommended by the National Policy Guidelines [21] include the following:

- Soaking the affected area in an antiseptic solution (e.g. Savlon, potassium permanganate, or hydrogen peroxide).
- Using flea repellents (e.g. Deet, Zanzarin, neem and coconut oil preparations, jojoba oil, or aloe vera extract).
- Surgical extraction at an appropriately equipped health facility.

### Treatments not listed in the guidelines

All treatment methods not listed in the guidelines. Manual and non-hygienic extractions are not recommended by the National Policy Guidelines [21].

## Methods

### Study design

A cross-sectional mixed-methods (quantitative and qualitative) study design was used. Quantitative data, such as socio-demographic information and KAP data, were collected using a questionnaire, while qualitative data were collected through semi-structured individual interviews with older adults and medical staff. Data were collected from April to June 2022.

### Study sites

This study was conducted in the Suba South and Ndhiwa sub-counties of Homa Bay County, western Kenya. The first sites were two community units (CUs) with the highest prevalence of tungiasis (JICA grassroots project survey, 2022). Next, three villages with the highest prevalence of tungiasis were selected from each sub-county of those CUs: Mwigika, Kibanga, and Rangiri from Kisaku CU: Kiemba B, Nyabwacheche, and Kibura C from Tonga Upper CU in the Suba South sub-county: Nyigino, Rota B, and Ndisi D from Upper Kayambo CU: and Korlango, Rachar, and Miyal from Kwamo CU in the Ndhiwa sub-county. The interviewed households are within 16–59 min of walking distance from the nearest health facilities (Suba South: 19–59 min: Ndhiwa: 33–49 min). However, the nearest health facilities are dispensaries and health centres, which are the lowest health facilities in Kenya.

### Sampling procedure

A purposive sampling method was used in this study. First, sample areas were identified in order of the most affected endemic area from the CU. Second, three villages from each CU with the highest tungiasis prevalence were selected. Community health volunteers (CHVs) who were well-known in the village, assisted with determining the household entry point to enable household number registration. The selected CHVs were those who regularly visited households to assess the members’ health condition and, at times, took them to health facilities.

CHVs assisted in screening the residents to determine their eligibility for participation. After a thorough explanation of the study objectives and procedures, the participants were contacted by CHVs and asked whether they were willing to participate in the study. Upon accepting to participate, the ‘informed consent’ form was read and explained to each participant, who then signed it.

### Sample size and study populations

#### Quantitative study

According to a previous household survey on treatment-seeking behaviour (JICA grassroots project survey, 2022), the estimated proportion of people who received non-listed treatments was 40%. This estimated sample size calculation was used for a two-sample comparison of proportions; for example, since educational background is an index for measuring poverty, it was used to calculate the sample size. The proportion of individuals with a high education level receiving non-guideline-listed treatments was estimated at 30%, and that of individuals with a low education level receiving non-guideline-listed treatments was assumed to be 50%, according to previous research [5, 12].

The sample size was computed as follows: alpha = 0.05 (two-sided), power = 0.8, p1 = 0.3, p2 = 0.5, n2/n1 = 1.00. Based on the 2.0 design effect together with incomplete or missing data, 5% of the data, reflecting 24 respondents, were excluded. Thus, 448 respondents were recruited for the study.

Four hundred and forty-eight residents were recruited for this study. They were all at least 18 years old, lived in the Suba South and Ndhiwa sub-counties and had personally experienced a *T. penetrans* infection in their lifetime. The questionnaire was read and filled in by trained research assistants. To avoid language bias, we translated the questionnaires into local languages according to the participants’ preferences then back-translated them. The survey was conducted door-to-door.

Based on Winter et al. [5] and Kimani et al. [23], the questionnaire used for the quantitative survey inquired about age, sex, marital status, occupation, educational level, knowledge of tungiasis, attitude towards tungiasis, tungiasis management practices, and experience with tungiasis. Regarding experience, we asked whether they had encountered tungiasis in the past and the kind of treatment options chosen or received.

#### Qualitative study

Thirty participants were sampled based on the inclusion criteria for the qualitative individual interviews. Twenty of the sampled respondents completed the questionnaire and were recruited for semi-structured interview A. Ten medical staff, particularly clinical officers and nurses, were recruited for semi-structured interview B.

Semi-structured interview A: We interviewed 20 participants (10 men and 10 women) aged 60 years and above (mean = 68, range = 60–94) who lived in the Suba South and Ndhiwa sub-counties and had personal experiences with *T. penetrans* infection. The mean interview time (range) for individual interviews was 10.87 (5.51–31.14) minutes. The older adult population was selected because they were: (1) considered a group with a high risk of tungiasis, and (2) most likely to have been treated for tungiasis during their lifetime. The interviews were carried out in the local language (Luo).

Semi-structured interview B: We also interviewed 10 participants (four clinical officers and six nurses) who worked in healthcare facilities in the most affected endemic area in the Suba South and Ndhiwa sub-counties and had experience with tungiasis treatment. All were recruited from different facilities. The mean age (range) of the participants was 35 (28–52) years. The mean interview time (range) for individual interviews was 8.49 (5.30–12.22) minutes. The interviews were carried out in English or Luo, according to participants’ preferences.

The guides for the semi-structured interviews, sourced from Schoonenboom and Johnson [24], included a list of treatment methods they used, the reason for the treatment selection, the best option for treatment, and the reason for considering each treatment option. The participants were interviewed by trained research assistants in a private room. Data were collected through both writing and voice recording. After the interviews, we confirmed that participants’ responses matched the information in their patient records in the facilities.

### Data analysis

#### Quantitative study

Respondents were categorised as either 'guideline treatment users' or 'non-guideline treatment users' according to the operational definition. If respondents selected both guideline and non-guideline treatments, they were categorised as non-guideline treatment users.

The obtained quantitative data were stored in Excel sheets and analysed using IBM SPSS Statistics ver. 25. A complete-case analysis was used. The use or non-use of guideline treatment was the dependent variable, while the following were the independent variables: No. of people living in the household; no. of children < 5 years; current infection status; age group; sex; marital status; occupation; education level; knowledge of tungiasis; perception of tungiasis; ashamedness; place for sleep; footwear of the respondent; footwear of the children; frequency of bathing; use of soap; frequency of sweeping the house; seeking health facilities; seeking traditional healer; avoid skin contact to prevent; wait and see to prevent; point of seeking treatment; and factors of treatment choices. Variables showing significance at p < 0.1 in univariate analysis were included in multiple logistic regression. Multiple logistic regression analysis was chosen because there is no time component and the dependent variable is binary. Factors that were significant at *p* < 0.05 were considered independently associated with choosing guideline-listed tungiasis treatments. Since this was an exploratory trial, no confirmatory testing was planned.

#### Qualitative study

Qualitative data were analysed using themes and used to triangulate the qualitative data. The two researchers coded the data independently. They then collaborated, through an iterative process, to group the data into categories and subcategories using the resulting codes. For semi-structured interview A, the categories were partially guided by Saunders’ theoretical framework [25].

We analysed the quantitative and qualitative data separately for all participants. To explain the results, an explanatory sequential design, in which the first phase of quantitative data collection and analysis is followed by the collection of qualitative data, was used for semi-structured interview A targeting older adults. Semi-structured interview B with clinical officers and nurses used a convergent parallel design, in which the quantitative and qualitative strands of the research are performed independently, and their results are brought together in the overall interpretation [24].

### Patient and public involvement

Patients and the public were not involved in the design of the study.

## Results

### Quantitative data

### Characteristics of study participants

Table 1 shows the socio-demographic characteristics of the participants. Of the total 448 adults recruited for the study, 421 agreed to participate and 410 completed the questionnaire (valid response rate: 91.5%). Regarding sex, 172 (42.1%) were male and 237 (57.9%) were female. The median age of the participants was 40 years. Furthermore, 90.7% of the participants (372) were farmers. Regarding education, 169 (41.2%) had dropped out of primary school, while 34.4% had completed primary education. The number of people using treatments listed in the guidelines was 188, and did not differ significantly based on sex, age, occupation, and education. However, a significantly higher number of respondents who were single, divorced, and widowed used treatments not listed in the guidelines compared to married respondents (*P* = 0.025).

**Table 1:** Characteristics of participants in the quantitative study

		Total (*n* = 421)		Treatment conforming with Guideline (*n* = 189)		Treatment not listed in Guideline (*n* = 232)		
		*n*	%	*n*	%	*n*	%	*p*
Sex	Male	179	42.5	84	44.4	95	40.9	0.489
	Female	242	57.5	105	55.6	137	59.1	
Age	18–29	82	19.5	27	14.3	55	23.7	0.136
	30–39	121	28.7	59	31.2	62	26.7	
	40–49	97	23.0	43	22.8	54	23.3	
	50–59	67	15.9	35	18.5	32	13.6	
	60≦	54	12.8	25	13.2	29	12.5	
Occupation*	House wife	11	2.6	4	2.1	7	3.0	0.128
	Farmer	383	91.0	178	94.2	205	88.4	
	Fishing industry	1	0.2	1	0.5	0	0.0	
	Small scale business	19	4.5	5	2.6	14	6.0	
	Formally employed	4	1.0	0	0.0	4	1.7	
	Others	3	0.7	1	0.5	2	0.9	
Education	None	36	8.8	18	9.5	18	8.6	0.392
	Some primary	176	42.9	79	41.8	97	41.8	
	Primary	142	34.6	64	33.9	78	33.6	
	Some secondary	27	6.6	15	7.9	12	5.2	
	Completed secondary or higher	40	9.8	13	6.9	27	11.6	
Marital status*	Single	9	2.1	1	0.5	8	3.4	0.024
	Married	358	85.0	170	90.4	188	81.0	
	Divorced	1	0.2	0	0.0	1	0.4	
	Widowed	53	12.6	18	9.5	35	15.1	
Treatments used**	Vaseline	177	42.0					
	Potassium permanganate	171	40.6					
	Self-extraction	114	27.1					
	Sodium carbonate	105	14.9					
	Coconut oil	99	23.5					
	Others	34	8.1					
	Missing data	8	1.9					

*Occupation and Marital status were tested using Fisher's exact test, and others were tested using the chi-square test

**This allowed for multiple answers. Others: Diazinon, other oils, paraffin, local herb

### Factors related to the selection of treatment

Out of 23 variables, 14 were found to be significantly correlated with guideline-listed treatment selection in simple logistic regression analysis; thereafter, multiple logistic regression analysis was performed. Logistic regression analysis revealed that ‘not knowing that fleas are the cause of tungiasis’ (Odds ratio [OR] = 0.31, 95% confidential interval [CI] 0.19–0.51, *P* < 0.001), ‘not seeking treatment from healthcare facilities’ (OR = 0.57, 95% CI 0.33–0.99, *P* = 0.046), ‘not seeking treatment from traditional healers’ (OR = 0.04, 95% CI 0.01–0.17, *P* < 0.001), and ‘wait and see to prevent disease in non-affected members’ (OR = 0.02, 95% CI 0.00–0.21, *P* = 0.001) were significantly correlated with using non-guideline-listed treatments for tungiasis (Table 2).

**Table 2:** Factors related to the selection of non-guideline treatments for tungiasis

Items		OR	Lower	Upper	*P*-value
Cause (ref. Correct: answering fleas)	Incorrect	0.31	0.19	0.51	< 0.001
Seeking the treatment to a health facility (ref. Going)	Not going	0.57	0.33	0.99	0.046
Seeking the treatment to a traditional healer (ref. Going)	Not going	0.04	0.01	0.17	< 0.001
Prevention methods for other non-affected members (ref. Do something)	Wait and see	0.02	0.00	0.21	0.001

OR, odds ratio; CI, confidence interval

### Qualitative data

### Characteristics of study participants

The qualitative study included 30 participants in two semi-structured interviews (Table 3). All participants clearly explained how they had treated their tungiasis.

**Table 3:** Characteristics of the participants in the qualitative study

	Older adults	Medical staff
Average (range) age	68 (60–94) years old	35.1 (28–52) years old
Sex	Male: 10; female: 10	Male: 7; female: 3
Sub-county	Ndhiwa: 10; Suba-South: 10	Ndhiwa: 5; Suba-South: 5
Designation		Clinical officers: 4; nurses: 6
Average time conducted	10.87 (5.51–31.14) minutes	8.49 (5.30–12.22) minutes

The following results were obtained according to the guidelines and are based on the responses to the question related to type of treatments used/recommended.

*Semi-structured interview A.* All the participants had used treatments not listed in the guidelines; 7 respondents mixed their treatments with guideline-recommended treatments, while 13 respondents solely used treatments not listed in the guidelines.

*Semi-structured interview B.* Four of the 10 medical staff interviewed used guideline-recommended treatments, and six respondents used treatments not listed in the guidelines.

### Tungiasis treatment options

A total of 28 tungiasis treatment options were identified in the qualitative study of both the older adults and medical staff (Table 4).

**Table 4:** Summary of the options for treating tungiasis

	Older adults (*N* = 20)	No. of answers	Medical staff (*N* = 10)	No. of answers
1	**Self-removal**	11	Surgical extraction	7
2	Potassium permanganate	5	Potassium permanganate	4
3	Petroleum jelly	4	**Lysol**	3
4	**Self-removal + paraffin**	4	**Iodine**	3
5	**Self-removal + petroleum jelly**	2	Savlon	2
6	**Gamatox**	2	Hydrogen peroxide	2
7	**Paraffin**	2	**Jik + iodine**	1
8	**Solanum incanum**	2	**Antibiotics**	1
9	**Menthol balm and Nyamtukru (local herb)**	2	Hydrogen peroxide + surgical extraction	1
10	**Diazinon**	2	**Surgical extraction + potassium permanganate**	1
11	**Self-removal +** **Solanum incanum**	2	**Calamine lotion**	1
12	**Self-removal + Aloe vera**	1		
13	**Self-removal + Diazinon**	1		
14	**Self-removal + Fermented cow milk**	1		
15	**Self-removal + Cow urine**	1		
16	**Diazinon + cow dung**	1		
17	**Cow dung**	1		
18	**Soda ash**	1		
19	**Grease**	1		

Normal text: guideline-listed treatments; bold text: treatments not listed in the guidelines

#### Use of guideline-listed treatments among older adults

Nineteen tungiasis treatment options were identified. However, only two of the 19 treatments (petroleum jelly and potassium permanganate) were listed in the guidelines. The participants indicated that their use of potassium permanganate was informed by CHVs and that sometimes the procedure was conducted in healthcare facilities. A man in his 60 s explained this stating, ‘*I came to know of it [potassium permanganate] when I went to the community health volunteer, who told me [about it]. I overheard people saying that jiggers are treatable*’.

#### Use of non-guideline-listed treatments among older adults

Eight of these included self-removal using pins, fish bone, hen’s feather, sisal plant, and other thorns from local plants such as Alaktar (*Acacia lahai*) and Ochuoga: Forssk [26]. The others included paraffin, Gamatox and Diazinon, organophosphorus insecticides. One participant in his 90 s explained that ‘*Gamatox helped people mostly*’, and an agrovet retailer issued him an organophosphorus insecticide as a treatment option for tungiasis. He was instructed to dilute the pesticide with an estimated volume of water, then soak his feet for one hour and pour the mixture into the floor holes and crevices suspected of flea infection. Much like those who used concentrated Jik and Lysol, this participant experienced unbearable skin irritation. Three respondents identified the use of cow’s milk, cow urine, and cow dung for tungiasis treatment. Furthermore, five responses were related to the use of some herbs and natural plants. A woman in her 60 s stated, ‘*The work of the cow dung is to prevent re-infection of other jiggers in that hole*’. Another two participants, a woman and a man, who were in their 80 s and 60 s, respectively, spoke regarding natural plants. The woman stated, ‘*Someone advised me to use Onyalobiro* (*Schkuhria pinnata*) [26]*, but it never worked. It is the doctors who helped me*’. The man stated, ‘*Ochok* (*Solanum incanum* L.) [27] *did not treat me fully. That was why I went to [the] hospital because Ochok only worked for two weeks and jiggers were back again’.*

#### Use of guideline-listed treatments among medical staff

Eleven tungiasis treatment options were identified in healthcare facilities, of which only five (surgical extraction, potassium permanganate, Savlon, hydrogen peroxide, and hydrogen peroxide + surgical extraction) are listed in the guidelines. A nurse in his late 20 s stated, ‘*We don’t have a standard measure… we are not sure* (about the mixing ratio)*, but we take around 3–5 L* (of water) *with 10 cc* (of hydrogen peroxide)’. Another clinical officer in his 30 s answered that a 0.1% mixture of potassium permanganate was prepared for tungiasis treatment. Another clinical officer in his 30 s answered that Lysol which is a product made with benzalkonium chloride, and it has been used as a medical disinfectant, is used as a 50% mixture.

#### Use of non-guideline-listed treatments among medical staff

All medical staff mentioned other products, except natural herbs or plants. Jik is a hypochlorous acid product, and it is recommended that it be diluted to 0.05% for use as household bleach. Three out of 10 interviews confirmed that interview responses and outpatient medical records matched. The other seven interview responses were not found, as those experiences were from different facilities before transferring.

### Reasons for the selection of treatment

For the older adults, the most frequently mentioned reason for selecting/using the treatments was ‘Someone else’s idea’, followed by ‘Availability and accessibility’ and ‘Problem resolution without professional help’ (Table 5). Furthermore, the most frequently mentioned reasons for selecting the best treatment were ‘effectiveness’, followed by ‘ease of use’, ‘safety’, and ‘availability’.

**Table 5:** Reasons for selecting a particular tungiasis treatment method

	Older adults		Medical staffs	
Reasons	Category	Sub-category	Category	Sub-category
Selecting/using the treatment	Someone else's idea (13)	Someone's advice (9) Saw someone's practice (2) Doctor's suggestion (1) Previous generation practice (1)	Supply situation (7)	Supply situation & availability (6) Affordability (1)
	Availability and Accesability (7)	Availability (6) Accesability (1)	Severity of the cases (4)	Severity of the cases (4)
	Problem resolution without professional help (4)	Own experience (4)	Consulting with others (3)	Consulting with others (2) Consulting public health (1)
			Don't know the guidelines (2)	Don't know the guide lines (1) Trial and error (1)
Selecting the best treatment	Effectiveness (22)	Good outcome (7) Killing jiggers (6) Fast response (3) Relieve itchiness and pain (3) No re-infection (2)	Effectiveness (10)	Good outcome (7) Fast response (3)
	Ease of use (2)	Ease of use (2)	Own experience (2)	Own experience (2)
	Safety (1)	Safety (1)	Availability (1)	Availability (1)
	Availability (1)	Availability (1)		

*Figures in parentheses are the number of people who answered

The most frequently mentioned reason by medical staff for selecting/using a treatment was ‘supply situation’, followed by ‘severity of the cases’, ‘consulting others’, and ‘don’t know the guidelines’. The most frequently mentioned reasons for selecting the best treatment were ‘effectiveness’, followed by ‘own experience’ and ‘availability’. A nurse who had treated tungiasis more than five times explained that she decided on the treatments through ‘trial and error’ (Table 5).

### Ease of accessing and using tungiasis treatments for older adults

**Fig. 1:**
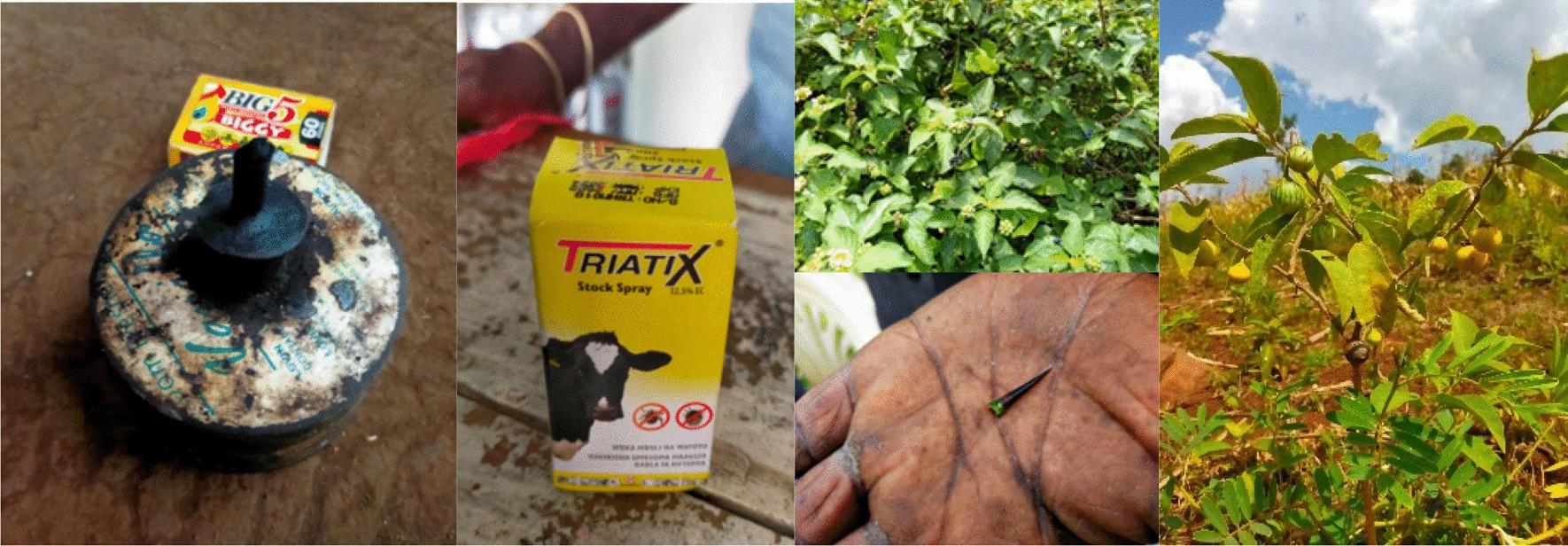
Pictures detailing local tungiasis treatment options (Left: Paraffin, typically obtained from their house; Middle: Organophosphorus insecticides from Agrobet; Right: Sisal thorn to be used as a natural extractor, Schkuhria pinnata “Onyalobiro”, and Solanum incanum “Ochok” obtained locally)

The most frequent treatment based on ease of use was paraffin, followed by thorns (Fig. 1). However, based on the ease of obtaining treatment, the most frequent treatment method was self-removal using thorns, followed by paraffin and *Solanum incanum*. The participants considered thorns as ‘not easy to use’ and Diazinon as ‘not easy to get’. Most respondents said that spending 0 ksh–10 ksh (1 ksh = 0.008 USD) on treatment was affordable, but spending 30 ksh–150 ksh on treatment was not.

## Discussion

### Treatments sought by older adults

This study found that some residents in tungiasis-endemic areas preferred the application of insecticides and hazardous chemicals for controlling sand flea infections. It also identified that self-extraction and application of local herbs and cow dung were considered most effective by the survey respondents; however, the efficacy of these products is not known. This is consistent with another study which found that some Northeastern Ugandans used engine oil, diesel, or petrol to treat tungiasis, and applied ash and tobacco after extraction [28]. The toxicity of these products, especially concentrated Jik or Lysol, which were commonly used by the respondents, have resulted in numerous skin irritations in humans [29] and death in domestic animals such as chickens and lambs [13]. This study demonstrated that few individuals consider or assess the harmful effects of these chemicals and insecticides on human skin and systems and on domestic animals.

### Factors affecting older adults’ choice of treatment and treatment-seeking behaviour

*Treatment availability, accessibility, and affordability.* In this study, the acceptance of given treatment method was influenced by the availability, accessibility, and affordability of familiar treatment options. This is in line with the findings of Elson et al. [15, 30], who observed that most patients in local communities prefer traditional healers due to easy access, continuous availability of their products, and the low costs involved. By contrast, in studies conducted in Brazil [5] and Uganda [28, 31]—which also show that manual self-extraction, antiseptics, oily preparations, ointments, and sometimes toxic substances were the most common tungiasis treatment options—extraction was the preferred treatment because participants believed that it was the appropriate treatment method, rather than due to its accessibility. This may be attributable to the difference in disease prevalence in the study area. Specifically, we identified a wider variety of non-guideline-listed treatment methods compared to other studies conducted in areas with high disease prevalence. For example, a study in Brazil [5] included data from areas with 30–50% prevalence rates, while the prevalence rates in the present study area were 3–5%. Because areas with lower disease prevalence rates offer less data, we cannot compare the number of choices for local treatments between more endemic and non-endemic areas. Nevertheless, the availability and accessibility of different treatment options remain essential parameters in determining the roles and uses of particular products in every society [21]. Concerning affordability, many surveyed residents, constrained by their economic and financial circumstances, resorted to risky treatment options that included applying cow urine or dung to open wounds, thereby risking secondary bacterial infections.

As most residents in tungiasis-endemic areas are farmers, strategies for improving the availability and affordability of effective treatment options, especially price setting for new drugs, should be aligned with the poverty index of the areas to enable most of the patients to afford the treatments. Therefore, crucial and urgent actions and policies are required to increase relevant resources including human and physical capacities within the local healthcare facilities.

*Lack of knowledge concerning cause of tungiasis.* Lack of knowledge about the cause of tungiasis was also correlated with using non-guideline-listed treatments for tungiasis. This result is inconsistent with those of previous studies conducted in Kenya and Uganda [23, 31], which suggested that residents in the tungiasis-endemic community were knowledgeable about it.

*Seeking assistance outside healthcare facilities.* Most residents reported seeking clarifications regarding tungiasis treatment from their previously infected peers and opted to employ the suggested treatment without further objection or inquiries on the related ill-health complications or side effects, showing poor health-seeking behaviours. This may be related to the finding that all the older adult participants were unaware of the guidelines and that the guidelines do not specify the appropriate use of treatments, indicating that the Kenyan guidelines for tungiasis treatment are unknown and untested in tungiasis-endemic regions. Additionally, one CHV told us that she referred a patient to the nearest hospital; however, when there was no medicine available there, the medical staff referred the patient back to the CHV (Personal communication from CHVs, 2022). Therefore, we can also surmise that health facilities are sometimes not effective in providing treatment for tungiasis patients, which discourages these patients from seeking treatment in the first place. Stigma may also be a barrier to patients seeking treatment outside the home. Some of the interviewed participants reported that using unprescribed herbs had limited success and prompted them to seek hospital interventions. Most of these participants initially self-treated with available and accessible therapeutic products and later sought treatment at healthcare facilities after their self-treatments failed or symptoms persisted. This predicts and presents the instances of mixed and multiple procedures involved in the treatment of tungiasis in endemic areas [32, 33]. This is in line with a study finding that reported that approximately 18% of acute and 8% of chronic diseases in rural Kenya were treated using multiple procedures or care providers [34]. The general healthcare-seeking behaviours of the residents in these local communities have been ranked, with systemic symptomatic and infectious diseases being the leading drivers of healthcare-seeking behaviours [2, 12]. A report on treatment-seeking behaviour in western Kenya for common infectious diseases [35] revealed that 88% of people sought treatment in any healthcare facility, and 48% sought care in a hospital. Compared to mild tungiasis cases, characterised by limited discomfort, infectious diseases present systemic symptoms such as fever, persistent pain, and diarrhoea, demanding therapeutic attention at different levels of medical facilities [2, 36].

Such incidents draw attention to the tendency of some community members in rural Kenya to consider specialists such as medical doctors. People listen to guidance received from non-medical persons, for example a clerk at a retail store, and follow these recommended treatments as though they were recommended by a doctor (Personal Communication from Healthcare Workers and Agrovet Workers, 2022). From this study, we understand that knowledge is insufficiently shared by national and county ministries of health, and the persuasiveness or lack of knowledge of commercial actors creates these gaps.

### Treatments used by medical staff

Based on this study’s findings, promoting Kenyan medical staff’s awareness of the national tungiasis treatment guidelines and updating these guidelines are important. Additionally, accurately and efficiently following the guidelines to treat tungiasis is crucial in both Kenya and elsewhere. Most medical professionals reported uncertainty about the exact treatment concentrations and dilutions required when treating tungiasis. For example, the recommended Lysol mixture for decontaminating a house is 2.5 oz (75 ml) to one quart of water (0.9 L), which is 7.7%, whereas the concentration used by medical professionals for skin treatment of tungiasis was 50%. Although a number of the medical staff reported the application of mixed methods for the control of tungiasis, some presented an unstable distribution of these methods to achieve the desired outcomes. This kind of insufficiency in knowledge among healthcare workers has been documented in a Nigerian study [37], particularly in the field of NTDs, as contributing significantly to poor healthcare outcomes. The study suggested that this knowledge gap among the medical staff has created disease identification difficulties and delayed the selection of appropriate treatment methods. Thus, improved training and awareness of the guidelines for healthcare workers are essential for eliminating this knowledge gap. Moreover, the guidelines need to be evidence-based and useful not only for medical staff, but also for affected community members.

Most medical staff stated that the selection of the most effective treatment options did not consider the safety measures involved. Therefore, random selections of the treatment options were based on previous outcomes, irrespective of the side effects involved. This highlights that a critical review of the healthcare system is urgently needed. Different stakeholders and policymakers, especially the Ministry of Health, need to establish a robust system for handling the safety aspects of different treatment methods. This calls for the training of local healthcare workers [20], standardisation of the local treatment programs, and review of patient safety before, during, and after tungiasis intervention programs.

### Recommendation and the limitations of the study

Based on the findings of this study, first, effective and safe treatments need to be made available to residents. Second, it is essential to investigate the efficacy and safety of the non-guideline-listed treatments that residents use to successfully treat tungiasis. Third, residents’ awareness of and knowledge about tungiasis and its treatment must be improved, and health facilities should be supplied with effective products and services to treat affected individuals and infested communities and households. For example, since the prevalence of tungiasis remains high among poor populations in many tropical countries, information on the affordability and availability of certified topical treatments, such as dimeticone oils [22] or neem and coconut oil preparations [30], needs to be provided to local populations through healthcare workers. Fourth, to reduce the disease burden in tungiasis-endemic areas, it is paramount that local healthcare providers and professionals are trained and that communities are empowered by educating them on tungiasis treatments, methods, and requirements. After further educating the medical staff and improving the capacity of health facilities managed by the Ministry of Health, we believe that communicating to the community that tungiasis treatments can be provided at health facilities will improve the residents’ treatment-seeking behaviours.

This research was likely limited by the stigma associated with tungiasis, which may have led patients to hide an infection or refrain from seeking treatment. In a study in Uganda [28], 54% of the respondents answered that they felt embarrassed when experiencing tungiasis. To prevent this, we asked CHVs who have relationships with the patients to collect data as research assistants. We conducted all examinations in a private room to make patients comfortable while answering questions.

Another limitation of this study is the time evolution of the treatments that the participants, especially the older adults, reported having used in the past. Most treatment methods mentioned by the respondents were not listed in the guidelines; therefore, additional research is needed to evaluate each treatment option for efficacy and safety. Further research is warranted to enhance policymaking and information dissemination.

## Conclusions

Residents’ socioeconomic, cultural, and accessibility factors and knowledge of medical staff were significant factors that influenced the residents’ treatment-seeking behaviours concerning tungiasis. This study provides baseline data for further investigation to establish an effective, safe, and sustainable treatment for tungiasis in western Kenya.

## Data Availability

The data used during analysis are available from the corresponding author upon reasonable request.

## Abbreviations

CHV: Community health volunteer
CU: Community unit
KAP: Knowledge, attitude, and practice
NTD: Neglected tropical disease
